# Workload Accomplished in Phase III Cardiac Rehabilitation

**DOI:** 10.3390/jfmk6020047

**Published:** 2021-05-28

**Authors:** Katrina L. Schultz, Carl Foster, Kimberley Radtke, Susan Bramwell, Cristina Cortis, Andrea Fusco, John P. Porcari

**Affiliations:** 1 Department of Exercise and Sport Science, University of Wisconsin-La Crosse, La Crosse, WI 54601, USA; schultzkatrina82@gmail.com (K.L.S.); kradtke@uwlax.edu (K.R.); sbramwell@uwlax.edu (S.B.); jporcari@uwlax.edu (J.P.P.); 2 Department of Human Sciences, Society and Health, University of Cassino and Lazio Meridionale, 03043 Cassino, Italy; c.cortis@unicas.it (C.C.); andrea.fusco@unicas.it (A.F.)

**Keywords:** cardiac rehabilitation, step count, sRPE

## Abstract

Exercise training is an important component of clinical exercise programs. Although there are recognized guidelines for the amount of exercise to be accomplished (≥70,000 steps per week or ≥150 min per week at moderate intensity), there is virtually no documentation of how much exercise is actually accomplished in contemporary exercise programs. Having guidelines without evidence of whether they are being met is of limited value. We analyzed both the weekly step count and the session rating of perceived exertion (sRPE) of patients (*n* = 26) enrolled in a community clinical exercise (e.g., Phase III) program over a 3-week reference period. Step counts averaged 39,818 ± 18,612 per week, with 18% of the steps accomplished in the program and 82% of steps accomplished outside the program. Using the sRPE method, inside the program, the patients averaged 162.4 ± 93.1 min per week, at a sRPE of 12.5 ± 1.9 and a frequency of 1.8 ± 0.7 times per week, for a calculated exercise load of 2042.5 ± 1244.9 AU. Outside the program, the patients averaged 144.9 ± 126.4 min, at a sRPE of 11.8 ± 5.8 and a frequency of 2.4 ± 1.5 times per week, for a calculated exercise load of 1723.9 ± 1526.2 AU. The total exercise load using sRPE was 266.4 ± 170.8 min per week, at a sRPE of 12.6 ± 3.8, and frequency of 4.2 ± 1.1 times per week, for a calculated exercise load of 3359.8 ± 2145.9 AU. There was a non-linear relationship between steps per week and the sRPE derived training load, apparently attributable to the amount of non-walking exercise accomplished in the program. The results suggest that patients in a community clinical exercise program are achieving American College of Sports Medicine guidelines, based on the sRPE method, but are accomplishing less steps than recommended by guidelines.

## 1. Introduction

Cardiac rehabilitation is of established benefit for decreasing the risk of future cardiovascular events [1,2,3], delaying/reversing the progression of atherosclerotic disease [4,5,6], reducing the rate of reinfarction and death [7,8,9], and accelerating the rate of recovery of functional capacity after clinical episodes [10,11]. The value of cardiac rehabilitation is evident even after accounting for contemporary revascularization and pharmacologic therapy [12]. Rehabilitation exerts its’ favorable effect, at least in part, through the very large effect of habitual exercise on both general, and cardiovascular, health [13,14,15,16]. Contemporary cardiac rehabilitation is accomplished through monitored exercise, education, and risk factor modification in phase II or phase III cardiac rehabilitation programs. Cardiac rehabilitation, and exercise training in general, decreases the risk of mortality and mitigates metabolic diseases [3], with the effect being somewhat dependent on the dose of exercise [3,6,13,14,15,16,17]. Further, the risk of developing new cardiovascular disease, and of mortality in relation to exercise, has been shown to be dose dependent [13,14,15,18].

Although there are widely accepted guidelines for exercising patients with known heart disease [19,20] and a rich literature on monitoring exercise training in athletes [21,22,23,24], there is virtually no evidence of how much exercise is actually accomplished by patients enrolled in rehabilitation programs. It can be argued that guidelines without evidence of how well they are complied with are of limited value. Accordingly, it seems desirable to have a practical method of quantifying the dose of exercise performed by cardiac rehabilitation patients, particularly in a way that gets beyond exercise mode specificity (e.g., steps per day).

One way to monitor exercise and physical activity is through step counts. Tudor-Locke et al. [18] found that individuals who averaged 5000 steps daily were more obese and had a higher risk of metabolic diseases, compared to individuals averaging 15,000 steps daily. Higher step counts were linked with an absence of obesity and decreased risk of disease in agricultural populations [25]. Recently, Kraus et al. [14] found that in individuals who increased daily steps, the risk of mortality decreased by ~10% for every additional 2000 steps per day. Recording participants’ daily step count is a simple method of estimating of how much exercise is done in one session, of how much physical activity is completed, outside of rehabilitation, and a practical way to prescribe and monitor exercise training. Indeed, Hambrecht et al. [6] presented evidence that regression of atherosclerotic lesions was associated with exercise at a level consistent with ~80,000 steps per week. Although step counts do not inherently address the very important aspect of exercise intensity, the strong dose dependency of the benefit of exercise seems to be present even when only step counts are considered [14,18,25]. Despite the presence of guidelines (generally of 7000–10,000 steps per day), to our knowledge there are no systematic data regarding step counts routinely performed during exercise programs primarily designed for secondary prevention.

However, step counts alone do not account for some modes of activity, such as cycling, Nu Step, rowing, arm cranking, and resistance training that are common elements of many rehabilitation programs. It would be useful if there were methods that could account for the multi-modal nature of most cardiac rehabilitation programs, as well as accounting for the net intensity of exercise training. Although accelerometry based methods have become popular, they still require patients to have access to and to wear an accelerometer [16].

The rating of perceived exertion (RPE) is a method of estimating internal training load and has been shown to be an acceptable surrogate of objective markers of exercise intensity such as percentage of heart rate (%HR) reserve and blood lactate accumulation either during an acute bout of exercise [26,27,28] or during an entire exercise session (thus including exercise duration which causes an upward drift in the workload-RPE relationship), the session RPE (sRPE) [21,22,29,30]. sRPE is easy to use and is accessible to most populations. Based on studies conducted by Foster et al. [22,29,30], Day et al. [31], and Herman et al. [32], sRPE has been shown to be valid in terms of evaluating entire training sessions at intensities ranging from light to vigorous, and in multiple modes of exercise [22]. Historically, sRPE was measured 30-min post exercise to get an accurate judgment of the overall intensity of the exercise training session. However, Christen et al. [33] and Foster et al. [22] have shown that sRPE is very temporally robust. Recent studies by Arney et al. [34,35] have demonstrated the interchangeability of the two most widely used RPE scales, further supporting the utility of sRPE for monitoring training. RPE is also inexpensive and “user-friendly,” which makes it ideal for cardiac rehabilitation populations. Further, the ability to use sRPE to “collapse across different modes of exercise” [22,30] makes it attractive for the often-multi-modal nature of cardiac rehabilitation programs. However, despite the generally accepted dose-response nature of responses to training in cardiac patients [6,17], to our knowledge there are no systematic data demonstrating the actual amount of training performed by cardiac patients using the sRPE approach.

Training load can be conceptualized as the amount of work done (duration x intensity) during an exercise bout [22]. Exercise training load has a demonstrated inverse relationship with mortality [3,4,13,15,36]. Total exercise related energy expenditure is also related to weight loss, decreases in abdominal fat, and reduction in waist circumference [17], which are frequent goals of cardiac rehabilitation therapy. Training load can be used to prescribe exercise and to personalize future exercise plans. This can be compared to the American College of Sports Medicine (ACSM) guidelines for 30 min of moderate intensity five times a week or 10,000 steps a day [14,19]. Accordingly, the purpose of this study was to document the training load accomplished by patients with known cardiovascular disease, or with risk factors likely to cause cardiovascular disease, in a community-based exercise program using both steps/day and the sRPE approach. Such data is uniquely needed relative to understanding how well contemporary programs execute professional society guidelines.

## 2. Materials and Methods

Thirty-two participants in the phase III La Crosse Exercise and Health Program (LEHP) were recruited. They ranged in age from 35–90, 62% were male and, typical of middle-aged Americans, they generally had elevated values for body mass index (BMI) (23–38). Twenty-two of the patients had experienced a documented cardiovascular event (myocardial infarction, revascularization surgery, angioplasty with stent), and the other 10 were >55 years of age and had risk factors for cardiovascular disease. Most of the patients had been treated for their primary medical problem at one of two local tertiary care hospitals, and had participated in Phase I and II cardiac rehabilitation programs there. They were self-referred to the LEHP as a venue to facilitate continuing exercise therapy, although individual consent from their own physicians was routinely secured. All of the patients were clinically stable, and the only contraindication to participation was clinical instability. Medications were conventional for this population (70% anticoagulants, 60% statins, 10% beta blockers, 12% ACE inhibitors/blockers, 32% anti hyperglycemic medicines) (Table 1). All participants provided written informed consent. The Institutional Review Board for the Protection of Human Subjects of the University of Wisconsin-La Crosse approved the study (Protocol #20-CF-081, 2020). The training program within the LEHP was scheduled to take place 3 times weekly. Although a 2.5 h block of open time was available at the facility, patients were instructed to participate for 1.0–1.5 h each time. Exercise training was intended to be performed primarily with aerobic activities that suited the subject. This could include walking, indoor cycling, arm-leg cycling, or swimming. Some light resistance training was performed by most of the participants. The exact mixture for each participant was highly individualized, primarily because their medical conditions and/or orthopedic history made some activities hard to perform. Most sessions included warm-up and cool-down calisthenics and stretching. We did not have access to maximal exercise tests on any of these patients, so regulation of exercise intensity was accomplished using RPE and the Talk Test.

Participants wore an ActiGraph device WGT3X-BT (Pensacola, FL, USA) for 3 weeks. It was worn on the hip, on the non-dominant side during all waking hours, except when bathing. Their daily step count was downloaded at the end of the study period and matched to program, and non-program, step counts based on day of week and time of day. They also tracked their exercise time and sRPE, using the classical 6–20 RPE scale, both during the LEHP, as well as during outside activities [22,29,30]. From this data, workload was calculated to determine the steps per day during the LEHP, during outside activities, the total for the day, and total for the week. Further, to determine training load using the sRPE method, the sRPE x duration in minutes both within the LEHP and in outside activities was computed. Data were collected over 3 consecutive weeks, as a convenient time interval, which is greater than the 2 weekdays and 1 weekend day used in classical epidemiologic studies. Further adding to the representativeness of the data collection period, one of the weeks contained a national holiday (Thanksgiving). The study was conducted during a period of the year when outside environmental temperatures were just above freezing (mid-Fall), although outside snow cover was minimal. One subject was removed due to loss of their ActiGraph device. Five subjects were removed due to either an inadequate amount of data (clear gaps in the recording) or improper recording on the activity monitor log sheet. Thus, a total of twenty-six participants were used in data analysis.

Steps per day and sRPE were presented as descriptive statistics and contrasted to recommended values for exercise training [19,20] and steps per day [14,18]. Statistical comparison of the mean exercise load versus recommendations was not appropriate. Regression statistics were compared between steps per day and the sRPE training load. Descriptive and regression statistics were computed using SPSS.

## 3. Results

The average number of total steps achieved was around 40,000 per week (Table 1), which is below the conventional benchmarks of 10,000 steps a day (50,000–70,000 steps weekly). About 80% of the steps were accumulated outside the LEHP. This suggests that more total exercise needs to be done to see an optimal decrease in risk of mortality [4,6]. The number of steps accumulated was greater on the days outside of the LEHP, probably because the activity at the LEHP was multi-modal, whereas almost all activity outside of the LEHP was accomplished by walking. The data did not allow fractionation of non-LEHP steps performed as “intentional exercise” versus “activities of daily living” On average, the subjects were exercising 4 days a week with the mean time of 260–270 min a week. This is above the recommended amount based on the ACSM guidelines [19]. The data also showed RPE to be higher in LEHP than during outside exercise (Table 2, Table 3, Table 4).

Time, RPE, sRPE derived training load and frequency accomplished in the LEHP are represented in Table 2. On average subjects spent about 160–170 min a week exercising with an RPE of about 12.5, which is just below the somewhat hard (RPE = 13) verbal cue widely taken as an idealized intensity for non-athletic individuals [37]. This gives an average workload of 2000–2100 AU (sRPE × minutes). Subjects attended the LEHP just under two times a week, out of the possible three times a week.

Time, RPE, sRPE derived training load and frequency performed outside of the LEHP are shown in Table 3. The average time spent exercising was 140–150 min a week, which is slightly less than the 160–170 min accomplished in LEHP. Average sRPE accomplished is 11.8, which is just above the “light” verbal cue, which is also slightly lower than the sRPE in LEHP. Similarly, the average load is about 1700–1800 AU which is slightly lower than the observed load in LEHP (2000–2100 AU). The frequency of exercising is about 2.4 times/week which is higher than the frequency of LEHP, indicating that participants are exercising more often, but for a shorter time and lower intensity on their own then they are in LEHP.

Table 4 presents the combined time, RPE, sRPE derived training load and frequency from both LEHP and outside exercise. The average frequency was 4.2 ± 1.5 times per week. The average duration was 260–270 min per week indicating that subjects, both in LEHP and outside are exceeding the recommended amount of weekly exercise (>150 min/week) in the ACSM guidelines [19]. Overall RPE averages 12.6 ± 3.84 which is just below the “somewhat hard” verbal cue, indicating that subjects are reaching moderate intensity level [37]. The sRPE Load was 3300–3400 AU per week. This is greater than the ~2000 AU recommended by the ACSM guidelines [19] of RPE = 13 × 30 min per day × 5 days per week.

**Figure 1:**
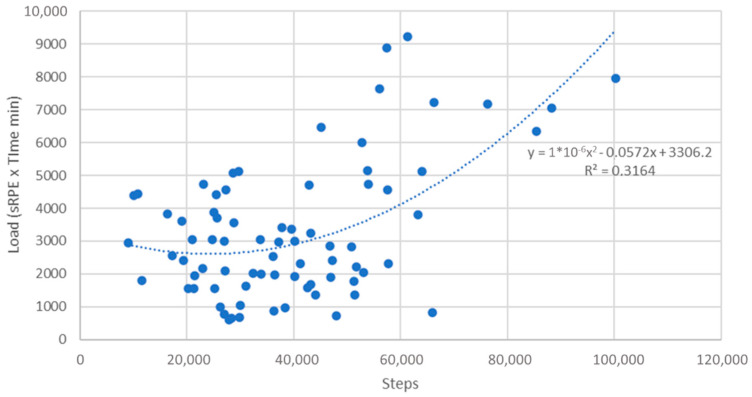
Relationship between total weekly step counts and session Rating of Perceived Exertion (sRPE) training load.

Figure 1 represents the relationship between total weekly steps and sRPE derived training load, with each week per subject being presented as a data point. The curvilinear line of best fit indicates that as step count increases there is an increase in sRPE derived training load. This graph shows that the majority of weekly averages are less than 60,000 steps and less than 5000 AU load. It also illustrates that a step counts <50,000 per week, there is relatively little change in sRPE based training load, probably because many of the steps were representative of activities of daily living, rather than structured exercise.

## 4. Discussion

Despite the unequivocal evidence of exercise-based rehabilitation programs in both rehabilitation and secondary prevention in patients with cardiovascular disease [1,2,3,4,5,6,7,8,9,10,11,12], physicians are still referring patients to programs in inadequate numbers. Potentially, this non-referral bias may be due to a poor understanding of how well exercise guidelines are implemented. The purpose of this study was to document the number of steps and sRPE derived training load accomplished both inside and outside a community-based Phase III clinical exercise program. These findings suggest that the recommended 10,000 steps/day (~50,000–70,000 steps per week) is not being met, but that the suggested dose of weekly exercise (>150 min/week) being accomplished exceeds the ACSM Guidelines [19]. There was a curvilinear trend between steps and load accomplished (Figure 1) demonstrating that, as step count increases, so does sRPE derived training load, at least after activity of daily living (ADL) steps (~35,000 steps per week) are accounted for. It also suggests that, at high training loads, there is an increased volume of multimodal exercise, which is not reflected by step count.

There were lower average steps observed in LEHP compared to outside, but overall higher time, sRPE, and load accomplished in LEHP. This indicates that these participants are working harder in the LEHP than during outside training. This supports the concept that patients should do more work, and work at higher intensity, in a community-based exercise program than they would otherwise do on their own. This matches general training practice guidelines for cardiac rehabilitation programs.

Currently there are no published data in regard to daily recording in any phase of a cardiac rehabilitation program. Assuming, on average, that participants exercise at a nominal walking pace of 5 kph, a rate requiring ~5.2 kcal per min (11.5 mL/kg × 90 kg = gross VO_2_ = 1.035 L/min, ×5 = ~5.2 kcal/min), the total of 266 min of exercise accomplished a week would equate to ~1282 kcal/week. Looking at only LEHP the average time accomplished was 162 min a week, which would equate to ~842 kcal/week. The outside exercise time per week averaged to 144 min, which would equate to ~749 kcal/week. According to Hambrecht et al. [6], 1400 kcal/week need to be accumulated in order to halt progression of coronary atherosclerotic lesions, and 2200 kcal/week needs to be expended to have regression in coronary lesions, although lower amounts of exercise are associated with an improved event free survival over 1-year compared to angioplasty + stenting [38]. The data shows that, on average, the participants are expending about 120 kcal/week less than what is needed to halt atherosclerosis. Assuming that one of the primary goals of exercise-based rehabilitation is secondary prevention, the current observations suggest that more total exercise needs to be accomplished.

An alternative to steps as a metric of exercise training load is the RPE, whether as a momentary index of training intensity, or an expression of the intensity x duration exercise load. Parfitt, Evans, and Eston [37] conducted a study on exercising for 30 min three times a week (90 min/week) for eight weeks at an RPE of 13 which would equal a load of 1170 AU (90 min/week × 13 RPE). This resulted in improvements in mean arterial pressure, total cholesterol, VO_2_max, and body mass index. Our subjects completed on average a workload of 2043 in LEHP and averaged a workload in total of 3360 AU, which is well above the Parfitt et al. [37] workload of 1170 AU, thus these same improvements would be expected to be observed in the current participants. Hambrecht et al. [38] conducted a randomized study that had subjects exercise 20 min daily. Assuming they worked at a similar RPE, their weekly load would be 1750 AU ((20 min × 7 days) × 12.5 RPE). He observed a higher event free survival of 88%, versus the 70% event free survival following percutaneous angioplasty with stenting (the nominal gold standard for patients with coronary artery disease). The total exercise load accomplished by our subjects is above that, plausibly accomplished, by Hambrecht et al. [38], suggesting the likelihood of good clinical outcomes. This is consistent with the nearly 50-year history of the LEHP, which has many participants with more than 20 years of participation, and with an experience of only ~1 untoward event per year.

This study does not address the total exercise workload accomplished in phase I and II cardiac rehabilitation, which address the rehabilitative goals of such programs in addition to secondary prevention. Given that one of the goals of the second part of phase II cardiac rehabilitation is secondary prevention, this information needs to be known. This study was conducted on a limited population sample, at a single community-based venue. Extending the results of this study to a larger population, at a larger number of venues, would provide more information about workload accomplished in rehabilitation programs. This is important, as Ades et al. [17] have suggested that the actual exercise load in conventional cardiac rehabilitation programs is inadequate to modify common risk factors that are targets of cardiac rehabilitation therapy.

While the Actigraph is a good device for calculating steps, there is a limitation with using multimodal equipment that may not be recognized by the device. This could be solved by computing MET × min [13] or MET × h [15] from chart records, or Time × RPE [22] from chart records, in established rehabilitation programs, to get a sense of the normal rate of progression of exercise load in Phase I and II rehabilitation programs, which typically include rowing machines, stationary cycling, Nu Step ergometers, and lifting weights. Data collection also happened to occur over the Thanksgiving holiday in Wisconsin. Due to celebration of the holiday and colder weather limiting exercise, this can result in decreased exercise levels that may not have occurred otherwise. Differentiating from outside activity or LEHP activity also provided some limitations. The best evidence is that nominally sedentary individuals accumulate about 5000 steps per day [14,18], but this likely varies greatly with age, season, and occupational demands. Being able to differentiate incidental versus intentional step counts would also be useful in further understanding the workload/health interaction in this population.

It would be beneficial to have this study replicated in phase I and II cardiac rehabilitation to know what workload is occurring at different phases. This information could be used to track if rehabilitation programs are doing enough exercise to accomplish their clinical goals and identify areas that could be improved. Replicating this study with more thorough education on how to use RPE, as well as properly fill out the log sheet, would help with participant error and more accurate data.

## 5. Conclusions

Our results in a community-based program showed that subjects achieved about 40,000 steps a week, which is less than the recommended 10,000 steps per day. Using an alternative method for calculating load by using sRPE, an average load of 3359 AU was achieved weekly. This information suggests that, while 10,000 steps may not be reached, adequate exercise, via different modalities that do not include steps, is indeed being achieved. Subjects averaged in total exercised 4.2 times weekly, with an average of 1.8 of those occurrences being at LHEP and average of 2.4 being outside of LEHP. The total average time spent exercising was 266 min a week, which is within the recommend amount of weekly exercise according to the ACSM Guidelines [19]. Comparing this study with the works of Hambrecht et al. [6,38] and Parfitt, Evans, and Eston [37], phase III cardiac rehabilitation can improve mean arterial pressure, cholesterol, body mass index, and increase chance of event free survival. However, the step count deficiencies observed here, compared to the regression data from Hambrecht et al. [6] suggest that more exercise could be profitable. Accordingly, community-based programs may benefit from having individuals exercise harder and longer than they otherwise would have on their own.

## Figures and Tables

**Table 1:** Mean and standard deviation of the steps per week achieved in La Crosse Exercise and Health Program (LEHP) and outside.

Week	LEHP (%)	Outside (%)	Total
1	7354 ± 6934 (17.9)	33,845 ± 15,662 (82.1)	41,199 ± 19,395
2	7483 ± 6342 (18.9)	32,308 ± 14,899 (81.7)	39,502 ± 17,939
3	6945 ± 5232 (17.9)	31,809 ± 15,529 (82.1)	38,753 ± 18,502
Average	7261 ± 6169 (18.2)	32,654 ± 15,360 (82.0)	39,818 ± 18,612

% Represent percentage of steps relative to total amount.

**Table 2:** Mean and standard deviation of the time (minutes), rate of perceived exertion (RPE), session RPE (sRPE), and frequency (times per week) for the La Crosse Exercise and Health Program.

Week	Time	RPE	sRPE	Frequency
1	173.5 ± 99.4	12.1 ± 1.9	2108.7 ± 1230.1	1.9 ± 0.8
2	157.9 ± 98.6	12.7 ± 1.9	2035.4 ± 1232.7	1.8 ± 0.7
3	155.8 ± 81.4	12.8 ± 1.9	1983.4 ± 1272.0	1.7 ± 0.6
Average	162.4 ± 93.1	12.5 ± 1.9	2042.5 ± 1244.9	1.8 ± 0.7

**Table 3:** Mean and standard deviation of the time (minutes), rate of perceived exertion (RPE), session RPE (sRPE), and frequency (times per week) for outside the La Crosse Exercise and Health Program.

Week	Time	RPE	sRPE	Frequency
1	146.9 ± 127.4	11.5 ± 5.7	1700.8 ± 1488.5	2.4 ± 1.4
2	142.1 ± 111.9	12.0 ± 5.8	1729.4 ± 1336.8	2.3 ± 1.5
3	145.7 ± 141.1	11.9 ± 5.8	1741.6 ± 1753.2	2.6 ± 1.5
Average	144.9 ± 126.4	11.8 ± 5.8	1723.9 ± 1526.2	2.4 ± 1.5

**Table 4:** Mean and standard deviation of the time (minutes), rate of perceived exertion (RPE), session RPE (sRPE), and frequency (times per week) for the La Crosse Exercise and Health Program and outside combined.

Week	Time	RPE	sRPE	Frequency
1	322.4 ± 181.8	12.6 ± 3.8	3617.0 ± 2188.4	4.3 ± 1.1
2	300.0 ± 163.8	12.8 ± 3.9	3273.2 ± 2248.0	4.2 ± 1.1
3	301.5 ± 166.9	12.4 ± 3.8	3189.2 ± 2001.2	4.2 ± 1.2
Average	307.3 ± 170.8	12.6 ± 3.8	3359.8 ± 2145.9	4.2 ± 1.2

## Data Availability

The data presented in this study are available on request from the corresponding author.
